# Association of Nutrition, Obesity and Skin

**DOI:** 10.3390/nu15092028

**Published:** 2023-04-23

**Authors:** Anja Saalbach

**Affiliations:** Department of Dermatology, Venereology and Allergology, University of Leipzig Medical Center, 04103 Leipzig, Germany; anja.saalbach@medizin.uni-leipzig.de

Nutrition, together with weight gain, alcohol consumption, physical activity, and other factors, is a risk factors for chronic inflammatory diseases. Dietary components, such as fatty acids, probiotics, saponins, fiber, antioxidants, and sodium, affect the inflammatory response and thus might contribute to the control, development, maintenance, and severity of chronic inflammatory diseases. Closely related to nutrition is the development of obesity. Several studies underline the association between the Western diet and obesity. Moreover, epidemiological studies demonstrate that obesity is related to the risk and severity of various chronic inflammatory disorders. Obesity is characterized by metabolic changes, such as hyperglycemia, dyslipidemia, hyperinsulinemia, a strong accumulation of adipose tissue, as well as a low-grade inflammatory status within the adipose tissue reflected by an increased expression of adipokines, chemokines, and cytokines. It is suggested that these changes affect both the homeostasis and activation of immune cells, resulting in the promotion of chronic inflammatory diseases and delayed wound healing. Inflammatory mediators from the adipose tissue, the adipokines, are discussed as links between obesity, adipose tissue accumulation, and inflammation severity.

New emerging data point out that nutritional factors play an important role in the control and restriction, as well as in the exacerbation and maintenance of inflammation. The goal of the present article collection, “Association of Nutrition, Obesity and Skin”, was to update current knowledge about the role of metabolic factors and nutritional components on skin inflammation and tissue repair.

Immune-mediated inflammatory skin diseases are characterized by a complex multifactorial etiology. Genetic and environmental factors interact in both the development and progression of the disease. The pivotal role of nutrition in inducing, exacerbating, or ameliorating several human diseases has been well documented. However, their relationship remains an open debate in the literature due to the complexity of the clinical course of immune-mediated skin diseases and the wide spectrum of the human diet. Therefore, it is essential to understand the mechanisms linking nutrition to inflammatory skin diseases, such as psoriasis, atopic dermatitis, hidradenitis suppurativa, bullous diseases, vitiligo, and alopecia areata. It is also important to know whether dietary changes can affect these diseases. Diotallevi et al. reviewed the role of diet in immune-mediated inflammatory skin diseases and discuss the usefulness of dietary interventions [1].

Skin and mucosal wounds comprise a broad variety of injuries, such as scars, pressure ulcers, diabetic skin ulcers, and burn wounds. The systematic review of Togo et al. summarizes data on the effect of oral or enteral probiotic therapy on skin or oral mucosal wound healing in humans. Seven studies, involving 348 individuals, were included in the study. In summary, the positive or neutral results observed do not suggest the effectiveness of probiotics for wound healing. The authors conclude that probiotics might be a promising field for future clinical research regarding the selection of probiotic strains, type of wounds, and target patient population [2].

Hidradenitis suppurativa (HS) is a chronic, recurrent, debilitating inflammatory skin disease of the hair follicle. It usually presents with lesions in the apocrine gland-bearing areas of the body, most commonly the axillary, inguinal, submammary, and anogenital regions. It is characterized by nodules, abscesses, fistulae, and permanent scarring. The quality of life of patients is severely compromised by these lesions. Lorite-Fuentes et al. performed a cross-sectional study, including 221 patients with HS. Disease severity, the adherence to a Mediterranean diet (MD), and the level of physical activity were documented. The authors show that greater adherence to a MD and more physical activity are related to lower HS severity. The authors conclude that MD, due to its anti-inflammatory action, might have additional benefits in the treatment of HS [3].

Obesity and metabolic syndrome are associated with increased incidence, disease severity, and reduced response to treatment of chronic inflammatory diseases, such as psoriasis. Pinter et al. describe an elegant study design to evaluate the impact of a dietary intervention targeting metabolic comorbidities on patient health status and psoriasis-specific outcomes. METABOLyx is a randomized controlled trial, which evaluates lifestyle intervention combined with secukinumab treatment in psoriasis. Patients with moderate to severe plaque psoriasis and metabolic syndrome were randomized into two groups, one receiving secukinumab the other one receiving secukinumab in combination with a lifestyle intervention program over 24 weeks in patients. METABOLyx represents the first large clinical trial of an immunomodulatory biologic combined with a standardized lifestyle intervention program [4].

Exposure to airborne particulate matter (PM) is an ever-increasing concern worldwide. In their original research article, Moen et al. demonstrate that PM increases intracellular oxidative stress, expression of pro-inflammatory cytokines, leptin, and dermal matrix metalloproteinases. In addition, melanin production was promoted by exposure to PM. Since exposure to air pollutants, including PM, is virtually unavoidable, the authors looked for new strategies to protect the skin from the harmful effects of environmental exposure to pollutants. The authors demonstrate that saponins within Korean red ginseng (KRG) extracts attenuate these effects. In conclusion, this study provides evidence that saponins possess multiphase anti-inflammatory, antioxidant, and antimelanogenetic effects on PM-exposed skin [5].

In summary, several dietary approaches, including supplementation, have been proposed to play a role in the pathogenesis, management, and/or therapy of chronic inflammatory skin diseases, such as psoriasis, atopic dermatitis, purulent hidradenitis, bullous diseases, vitiligo, and alopecia areata. In some cases, it may be helpful simply to avoid the triggers that have been identified. In others, supplements and dietary changes may be worth considering. However, further research into dietary manipulation and the effect of dietary components on these skin conditions is needed to better understand and treat patients.
